# Editorial: Advances in tick-borne diseases

**DOI:** 10.3389/fcimb.2025.1630872

**Published:** 2025-06-13

**Authors:** Omid Teymournejad, Aditya Kumar Sharma, Deepak Kumar

**Affiliations:** ^1^ Department of Pathology, College of Medicine, University of Illinois at Chicago, Chicago, IL, United States; ^2^ Office of Disease Control, Division of Laboratories, Illinois Department of Public Health, Chicago, IL, United States; ^3^ Department of Life Sciences, School of Biosciences and Technology, Galgotias University, Greater Noida, Uttar Pradesh, India; ^4^ Center for Molecular and Cellular Biosciences, School of Biological, Environmental, and Earth Sciences, University of Southern Mississippi, Hattiesburg, MS, United States

**Keywords:** tick-borne diseases, severe fever with thrombocytopenia syndrome (SFTS), multi-epitope vaccine, metalloprotease BcEnhancin, tick *Ixodes ricinus*, *Bartonella henselae*

Tick-borne diseases have a long history, which begin with Victor Babes description of Texas cattle fever in 1888 caused by the protozoan parasite *Babesia bigemina* and transmitted by the tick *Rhipicephalus annulatus* (Vannier et al., 2015). In 1893, Theobald Smith and Frederick Kilborne provided the first experimental proof of tick-mediated transmission by demonstrating that *Rhipicephalus annulatus* ticks transmitted a protozoan parasite *Babesia bigemina* to cattle, establishing our foundational understanding about vector-borne disease (Vannier et al., 2015). In 1906, Rocky Mountain spotted fever, caused by *Rickettsia rickettsii* and transmitted by the Rocky Mountain wood tick (*Dermacentor andersoni*), was identified as the first recognized tick-borne rickettsial disease, marking a significant milestone in the advancement of vector biology research (Walker, 1985; Liu, 2015).

**Figure 1:**
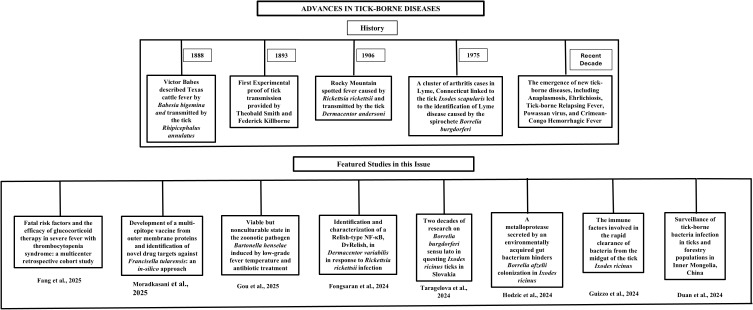
Flowchart illustrating a brief history of tick-borne diseases and content of the editorial.

In 1975, a cluster of arthritis cases in Lyme, Connecticut linked to the tick *Ixodes scapularis* (previously *Ixodes dammini*) led to the identification of Lyme disease caused by the spirochete *Borrelia burgdorferi*. Lyme disease presents with symptoms ranging from fever and rash to severe joint and neurological complications if untreated, and it remains the most prevalent tick-borne disease in the United States, with approximately 500000 cases annually and an economic burden exceeding $1 billion (Williamson and Calabro, 1984; Anderson and Magnarelli, 1994). Recent decades have seen the emergence of new tick-borne diseases, including Anaplasmosis, Ehrlichiosis, Tick-borne Relapsing Fever, Powassan virus, and Crimean-Congo Hemorrhagic Fever (Ismail et al., 2022; Sharma et al., 2023). These diseases pose significant challenges due to gaps in understanding their biology, diagnostics and treatment. This editorial highlight research published in the Research Topic “*Advances in tick-borne diseases*.” (**Figure 1**).

The Research Topic begins with a multicenter retrospective cohort study by Fang et al., which aimed to identify risk factors associated with fatal outcomes and evaluate the efficacy of glucocorticoid therapy in patients with Severe Fever with Thrombocytopenia Syndrome (SFTS), a viral tick-borne disease. Using regression analyses and nomograms, Fang et al. identified old age, consciousness disturbance, low monocyte count, prolonged activated partial thromboplastin time and high viral load as predictors of fatal outcome of SFTS. The research paper concluded that high-dose glucocorticoid (GC) therapy should be used cautiously as relatively high doses and early GC therapy increase mortality in SFTS patients, while low-dose GC treatment in patients with severe symptoms and low aspartate aminotransferase levels improved survival. This predictive model aids in risk stratification and guides tailored GC therapy for SFTS patients.

In another work, Moradkasani et al. employed *in silico* strategies to develop a multi-epitope vaccine (MEV) against *Francisella tularensis*, a highly infectious tick-borne bacterium. Using subcellular localization tools and reverse vaccinology pipelines, Moradkasani et al. screened 1921 proteins and identified 12 vaccine candidates. In this study, authors proposed MEV designed for seven immunodominant epitopes from four outer membrane proteins (including two hypothetical proteins, an OmpA family protein, and PD40), which each having properties including increased antigenicity, solubility, thermostability and half-life. Additionally, the study proposed 10 novel drug targets involved in translation, methylation and porphyrin biosynthesis, providing broad treatment options against various *F. tularensis* strains and addressing limitations in current tularemia treatments.

Similarly, Fongsaran et al. characterized *DvRelish*, a Relish-type NF-κB transcription factor in the tick *Dermacentor variabilis*, and its role in immune defense against *Rickettsia* infection. The authors found an increased *DvRelish* expression in tick hemocytes during *Rickettsia* infection compared to control. Also, RNA interference-mediated knockdown of *DvRelish* led to an increased rickettsial loads at 48 hours post-infestation, underscoring the role of *DvRelish* in the tick immune response. These findings highlight *DvRelish* as a central regulator of tick immunity and suggest that NF-κB signaling significantly influences vector competence for *Rickettsia* spp.

Further, Gou et al. demonstrated that *Bartonella henselae*, a zoonotic tick-borne bacterium, evades host cell responses due to its ability to enter into a viable but nonculturable (VBNC) state under stress conditions, such as fever (38.8°C for 19 days) or antibiotic exposure (4 days). Studies demonstrated that, despite losing culturability, VBNC cells remained viable and retained intact cellular structures, as evidenced by transmission electron microscopy. Notably, the proteomic data demonstrated that the proteins associated with host cell invasion and stress resistance are upregulated and proteins related to cell signaling and cellular processes were downregulated. Studies also showed that *Bartonella henselae* can survive hostile conditions by entering a VBNC state and this state contributes to drug resistance, host invasion, diagnostic and treatment challenges.

Next, Taragelova et al. summarized a 20-year surveillance study (1999–2019) of *Ixodes ricinus* ticks across 16 locations in Slovakia, testing 17,249 questing ticks for *Borrelia burgdorferi* sensu lato (s.l.). The key findings of the study include an overall prevalence of 18.8%, with 15.1% of nymphs and 24.3% of adults testing positive. Nine species within the *B. burgdorferi* s.l. complex were identified, including *Borrelia afzelii*, *B. garinii/B. bavariensis*, *B. valaisiana, B. lusitaniae*, *B. burgdorferi* sensu stricto, *B.* sp*ielmanii, B. bissettii*, *B. kurtenbachii*. Out of these *Borrelia afzelii* (37.1%), *B.garinii/B. bavariensis* (24.7%), and *B. valaisiana* (15.4%) were the most frequent and found at all the study sites whereas *B. lusitaniae*, *B. burgdorferi* sensu stricto, *B. bavariensis* and *B.* sp*ielmanii* were found in four samples and *B. bissettii* and *B. kurtenbachii* were also recorded in single cases. Additionally, the infection rate of *Borrelia burgdorferi* s.l. varied across habitats, with the highest prevalence observed in natural environments and the lowest in urban settings. These findings underscore the importance of long-term surveillance to evaluate the influence of climatic and socioeconomic factors on the distribution of tick-borne diseases.

Towards understanding the significance of gut microbiota in determining tick vector competence and defining the vector competence, Hodzic et al. revealed that *Bacillus cereus* LTG-1, an environmentally acquired gut bacterium in *Ixodes ricinus*, impairs *Borrelia afzelii* colonization through a secreted metalloprotease referred as *BcEnhancin*. Oral administration of *B. cereus* LTG-1 or purified *BcEnhancin* degraded the glycan-rich peritrophic matrix in the tick gut, which reduce *B. afzelii* load. Additionally, qRT-PCR data revealed that *myd88* and *peritrophin 1* genes were upregulated after 48 h of administration of recombinant BcEnhancin. Both genes are linked to immune system and peritrophic matrix in the tick gut respectively. These findings highlight the tick gut microbiome’s role in shaping vector competence and offer new insights into the interactions among *Borrelia*, ticks, and their resident microbes.

In a similar direction, Guizzo et al. investigated the immune response of *Ixodes ricinus* ticks following exposure to either the Gram-positive bacterium *Micrococcus luteus* or the Gram-negative *Pantoea* sp. Although the tick midgut harbors a limited and inconsistent microbiota about which our understanding remains incomplete, the ticks were able to rapidly clear artificial infections with both *M. luteus* and *Pantoea* sp. Transcriptomic and proteomic analyses revealed several constitutively expressed antimicrobial peptides including defensins, amidase effectors, lysozymes, and gamma interferon-inducible lysosomal thiol reductases (GILTs). Antimicrobial activity assays confirmed that defensins 1 and 8 are highly effective against Gram-positive bacteria such as *Micrococcus luteus*. These findings suggest that a pre-existing and multi-component antimicrobial system in the tick midgut plays a central role in rapidly eliminating invading microbes.

Lastly, the editorial concludes with the study by Duan et al., who investigated tick-borne bacteria and their associated infections in Arxan, Inner Mongolia, China, by analyzing 282 *Ixodes persulcatus*, 13 *Dermacentor silvarum* ticks, and 245 human blood samples. Using 16S rDNA sequencing and species-specific PCR, the authors detected *Candidatus Rickettsia tarasevichiae* (89%) and *Borrelia garinii* (17%) as the most prevalent pathogens in *I. persulcatus*, with 13% coinfection. In human samples, *B. garinii* (4.9%), *Rickettsia slovaca* (0.82%), and *Coxiella burnetii* (0.41%) were detected, with seroprevalence for spotted fever group rickettsiae (SFGR) and *B. burgdorferi* at 5.71% and 13.47%, respectively. The study confirmed *B. garinii* transmission from ticks to humans and reported the first detection of *B. miyamotoi* (7%) in ticks and *R. slovaca* (0.82%) in humans in Arxan, highlighting the need for ongoing surveillance.

Overall, we believe that collection of research articles featured in this Research Topic advance our understanding of tick-borne diseases by elucidating pathogen biology, vector competence, and host immune responses. From predictive models for SFTS treatment to innovative vaccine strategies for *Francisella tularensis* and insights into tick microbiome interactions, these findings pave the way for improved diagnostics, therapeutics, and prevention strategies. As tick-borne diseases continue to emerge and expand due to environmental and climatic changes, sustained research and surveillance are critical to mitigate their global public health impact.
